# Chicago Pasteur Institute

**Published:** 1893-12

**Authors:** 


### CHICAGO PASTEUR INSTITUTE.

Dr. A. Lagorio, Director of the Chicago Pasteur Institute, begs leave to inform you of the results of the preventive inoculations against hydrophobia attained at this Institute since its inauguration, July 2 1890.

To date 302 persons have been treated, classified as follows:

104 bitten by animals recognized and ascertained to be rabid by the experimental proof made in the laboratory; or by the death of other persons or animals bitten by the same animal.

126 bitten by animals recognized to be rabid by the symptoms of the disease shown during life.

72 bitten by animals strongly suspected to be rabid.

282 persons were bitten by dogs, 7 by horses, 7 by cats, 3 by skunks, 2 by wolves, 1 by a mule.

One death was reported among the patients treated, thus giving a mortality of only 0.33 per cent.

